# Class 1 Histone Deacetylases and Ataxia-Telangiectasia Mutated Kinase Control the Survival of Murine Pancreatic Cancer Cells upon dNTP Depletion

**DOI:** 10.3390/cells10102520

**Published:** 2021-09-23

**Authors:** Alexandra Nguyen, Melanie Dzulko, Janine Murr, Yun Yen, Günter Schneider, Oliver H. Krämer

**Affiliations:** 1 Department of Toxicology, University Medical Center, Obere Zahlbacher Str. 67, 55131 Mainz, Germany; alexandra.nguyen@uni-mainz.de (A.N.); medzulko@uni-mainz.de (M.D.); 2 Medical Clinic and Polyclinic II, Klinikum rechts der Isar, Technical University Munich, 81675 München, Germany; janine.murr@tum.de (J.M.); guenter.schneider@tum.de (G.S.); 3 Ph.D. Program for Cancer Biology and Drug Discovery, Taipei Medical University, 250 Wu Hsing Street, Taipei 110, Taiwan; yyen@tmu.edu.tw; 4 Department of General, Visceral and Pediatric Surgery, University Medical Center Göttingen, 37075 Göttingen, Germany

**Keywords:** apoptosis, ATM, cancer, DNA damage, HDAC, PDAC cells, replication stress, RNR

## Abstract

Pancreatic ductal adenocarcinoma (PDAC) is a highly aggressive disease with a dismal prognosis. Here, we show how an inhibition of de novo dNTP synthesis by the ribonucleotide reductase (RNR) inhibitor hydroxyurea and an inhibition of epigenetic modifiers of the histone deacetylase (HDAC) family affect short-term cultured primary murine PDAC cells. We used clinically relevant doses of hydroxyurea and the class 1 HDAC inhibitor entinostat. We analyzed the cells by flow cytometry and immunoblot. Regarding the induction of apoptosis and DNA replication stress, hydroxyurea and the novel RNR inhibitor COH29 are superior to the topoisomerase-1 inhibitor irinotecan which is used to treat PDAC. Entinostat promotes the induction of DNA replication stress by hydroxyurea. This is associated with an increase in the PP2A subunit PR130/PPP2R3A and a reduction of the ribonucleotide reductase subunit RRM2 and the DNA repair protein RAD51. We further show that class 1 HDAC activity promotes the hydroxyurea-induced activation of the checkpoint kinase ataxia-telangiectasia mutated (ATM). Unlike in other cell systems, ATM is pro-apoptotic in hydroxyurea-treated murine PDAC cells. These data reveal novel insights into a cytotoxic, ATM-regulated, and HDAC-dependent replication stress program in PDAC cells.

## 1. Introduction

Pancreatic ductal adenocarcinoma (PDAC) is an unresolved clinical problem with a five-year survival rate below 10%. This dismal prognosis is also of concern because PDAC is estimated to rise from the fourth to the second leading cause of cancer-related deaths by 2030 [1,2,3]. Thus, additional treatment options for this aggressive, often drug resistant, and highly metastatic tumor of the digestive system are required.

Histone deacetylases (HDACs) are epigenetic modulators that fall into four classes. Histone deacetylase inhibitors (HDACi) are currently tested in several clinical trials and four HDACi have been approved for the treatment of leukemic diseases [4,5]. HDACi have also been tested as drugs to treat PDAC cells [6,7,8]. It turned out that HDAC2 promoted the survival and robustness of PDAC cells against the topoisomerase-2 inhibitor etoposide and the tumor necrosis factor-related apoptosis-inducing ligand TRAIL. The underlying mechanism is a suppressive effect of HDAC2 on the pro-apoptotic factors NOXA and TRAIL receptor-1/DR5 [9,10]. Accordingly, inhibition of class 1 HDACs and induction of the proteasomal degradation of HDAC2 by the HDACi valproic acid [11] sensitizes PDAC cells to etoposide and TRAIL [9,10]. Gemcitabine, a nucleoside analog and ribonucleotide reductase (RNR) inhibitor, is used in the clinic to treat PDAC [12]. The HDACi 4-phenylbutyrate, AR-42, romidepsin, and ACY-1215 suppress the proliferation and viability of PDAC cells and combine favorably with gemcitabine against such cells in culture and in xenograft models [13,14,15]. Similar data were found when gemcitabine was given with the HDACi belinostat, vorinostat, and trichostatin-A to PDAC cells in vitro and in vivo [16,17]. HDAC3 is linked to the development of smoking-induced PDAC through regulation of the cytokine IL-6 and interactions between cancer cells and tumor promoting macrophages [18]. The new HDACi AES-135 targets HDAC3, HDAC6, HDAC11, and is effective against PDAC cells in vitro and in mice [19]. Other HDACs, including HDAC1, HDAC7, and HDAC8 are likewise necessary for the survival of such tumor cells [17]. Genetic experiments found that HDAC1, HDAC2, and HDAC6 could modulate the synergistic killing of PDAC cells by HDACi and gemcitabine [15]. In addition to a modulation of pro- and anti-apoptotic proteins, structurally divergent HDACi alter cytoskeletal organization and a dysregulation of energy control, nucleotide metabolism, and DNA repair protein expression [17,20,21,22]. Moreover, the FDA-approved HDACi panobinostat and valproic acid promote the killing of PDAC cells by natural killer cells [23].

In a phase-1 study, 21 patients with non-metastatic PDAC received vorinostat, irradiation, and capecitabine (the pro-drug of the anti-metabolite 5′-fluoruracil). This regimen was well-tolerated and achieved stable disease in 19 patients [24]. Another phase 1/2 study enrolled 25/22 patients with PDAC. These patients received gemcitabine and the HDAC1/HDAC2/HDAC3/HDAC11 inhibitor mocetinostat. This trial with a small number of patients had to be ceased due to significant toxicity [25]. Nonetheless, several studies illustrate that HDACs are key targets in PDAC.

HDACi combine favorably with other drugs that target epigenetic modifiers. A combined targeting of epigenetic readers of the bromodomain and extraterminal domain (BET) protein family and HDACs with the HDACi SAHA suppresses PDAC development and growth in mice. This requires the cell cycle dependent kinase inhibitor (CDKi) p57 [26]. Consistent herewith, a fusion molecule of the BET inhibitor JQ1 and the class 1 HDACi CI994 impairs the proliferation of PDAC cells [27] and BRD4 locates to genes that are induced upon HDAC inhibition in PDAC cells [28]. The lysine demethylase-6A (KDM6A) also determines the sensitivity of PDAC cells to HDACi. The overexpression of KDM6A and its elimination by CRISPR-Cas9 antagonistically modulate the expression of tumor suppressors in PDAC cells, including the CDKi p21. A complex of KDM6A and the histone acetyltransferase p300 positively regulates the acetylation of histone H3 at K27 and consequently the expression of p21. Vorinostat can overcome the loss of p21 expression in KDM6A null cells and this is associated with a growth suppression of PDAC cells in mice [29].

Hydroxyurea is a specific and reversible inhibitor of the RRM2 subunit of RNR. RNR catalyzes the synthesis of dNTPs for DNA replication during S phase [30]. Combinations of hydroxyurea and HDACi additively to synergistically induce apoptosis of solid tumor and leukemic cells [31,32,33,34,35,36,37,38]. In several cancer cells, this beneficial effect relies on a disruption of the hydroxyurea induced crosstalk between the transcription factors NF-κB p65 and p53 by HDACi [39,40]. Mutant p53 also enhances anti-apoptotic NF-κB target gene expression. In PDAC cells with mutant p53, HDAC1, HDAC2, and HDAC3 promote the expression of the anti-apoptotic protein survivin. This is due to a regulation of the transcription factor MYC which induces transcription of the mutated *p53* gene [34,41]. In other tumor cell types, the cooperative induction of apoptosis by hydroxyurea and HDACi depends on a decrease of the CDKi p21 and p27 [33,35], the reduced expression of the receptor tyrosine kinase EGFR, an increase in the pro-apoptotic BCL2 protein BIM [42], and a breakdown of checkpoint kinase signaling and cell cycle control due to an upregulation of the mRNA and protein expression of the protein phosphatase 2A (PP2A) subunit PR130, which is also known as PPP2R3A [32].

Using four genetically defined murine PDAC cells we analyzed how hydroxyurea and entinostat, a specific inhibitor of the class 1 HDACs HDAC1/HDAC2/HDAC3 [43], affect this tumor type. We additionally tested how the topoisomerase-1 inhibitor irinotecan [3] and the new RNR inhibitor COH29 [44,45] affected these cells. We reveal that PDAC cells are very sensitive to an inhibition of RNR. Despite accentuated DNA damage upon HDAC inhibition during hydroxyurea-induced replication stress, class 1 HDACs are necessary for the activation of ATM and apoptosis induction.

## 2. Materials and Methods

### 2.1. Drugs and Chemicals

Entinostat and KU-60019 were purchased from Selleck Chemicals, Munich, Germany; hydroxyurea, irinotecan, and propidium iodide (PI) were from Sigma-Aldrich Chemie GmbH, Munich, Germany; annexin V-FITC-conjugated was from Miltenyi Biotec, Bergisch Gladbach, Germany. The synthesis of COH29 was recently described in [44].

### 2.2. Murine PDAC Cell Lines

The isolation and culturing of primary pancreatic tumor cells lines were described [46,47]. The lines used in the study were obtained from a recently characterized murine PDAC cell line cohort [48] and are all driven by K-RAS^G12D^ (KC cell lines dependent on the *LSL-Kras^G12D^* [49] or *FSF-Kras^G12D^* [50] alleles). The following lines were used: S821 (genotype: Pdx-Flp +/−, FSF-Kras +/−, R26-FSF-CreERT2 +/+), 8296 (genotype: p48-Cre +/−, LSL-Kras +/−), 8248 (genotype: p48-Cre +/−, LSL-Kras +/−; TVA +/+), and S411 (genotype: Pdx-Flp +/−, FSF-Kras +/−); further details can be found in the references [46,47]. Cells were cultured in high glucose DMEM medium (D5796, Sigma-Aldrich Chemie GmbH, Munich, Germany), containing 10% fetal calf serum (FCS) (Sigma-Aldrich Chemie GmbH, Munich, Germany, or Pan Biotech, Aidenbach, Germany) supplemented with 1% (*w*/*v*) penicillin/streptomycin (Sigma-Aldrich Chemie GmbH, Münich, Germany).

### 2.3. Immunoblot

Immunoblots were carried out as described by us recently [4,51]. Membranes were blocked in 5% milk and antibodies were diluted in 2% milk (cow milk powder was purchased from Carl Roth, Karlsruhe, Germany, and diluted in TBS-Tween-20) [4]. Antibodies were from Abcam, Cambridge, UK, p21 (ab109199), 1:1000, BD Bioscience, Heidelberg, Germany, cleaved PARP (552596), 1:500, Cell Signaling, Frankfurt/Main, Germany, ATM (cs-2873), 1:500, CHK1 (cs-2360), 1:500, p-CHK1 (S317) (cs-12302S), 1:1000, p-CHK1 (S345) (cs-2348S), 1:1000, p-CHK1 (S296) (cs-90178S), 1:1000, CHK2 (cs-2662S), 1:1000, cleaved caspase 3 (cs-9661), 1:500, PP2A-A (cs-2039), 1:1000, PP2A-C (cs-2259), 1:1000), Novocastra Leica Biosystems, Wetzlar, Germany, p53 (NCL-p53-CM5p), 1:500, Novus Biologicals, Heidelberg, Germany, p-KAP1 (S824) (NB100-2350), 1:1000, PPP2R3A (NBP1-87233), 1:1000, Rockland/Biomol, Hamburg, Germany, p-ATM (S1981) (200-301-400), 1:1000, Thermo Fisher Scientific, Frankfurt/Main, Germany, RRM2 (PA5-13570), 1:1000, and Santa Cruz, Heidelberg, Germany, β-actin (sc-47778), 1:5000, HSP90 (sc-13119), 1:5000, ɣH2AX (S193) (sc-101696), 1:1000, RAD51 (sc-8349), 1:1000.

### 2.4. Flow Cytometry

Cell death and cell cycle distribution were determined using flow cytometric analysis [4,51]. For cell cycle distribution analysis, cells were detached from cell culture dishes with trypsin/EDTA and collected with the medium in a FACS tube. After 5 min of centrifugation at 1300 rpm the cell pellets were washed with PBS and fixed with 80% EtOH at −20 °C overnight. Cells were incubated with 330 µL Ribonuclease A (Carl Roth, Karlsruhe, Germany; final concentration 20 µg/mL) for 1 h at room temperature and subsequently stained with PI (final concentration 12.5 µg/mL). For apoptosis analysis the cells ware harvest as described above. After washing the pellets with PBS, cells were stained with annexin/V for 20 min at room temperature. After PI staining the samples were measured immediately with a FACSCanto Flow Cytometer and analysis was performed with the FACSDIVa^TM^ Software (BD Biosciences, Heidelberg, Germany). 

### 2.5. Statistics

Statistical analyses were carried out using one- and two-way ANOVA from GraphPad Prism Vers.8.3.0. Correction for multiple testing was achieved with Bonferroni multiple comparisons test. As a measure of significance, *p* values are indicated. When we noted differences that were not statistically significant, we termed them trends. These did not obtain asterisks.

## 3. Results

### 3.1. Class 1 HDACs Promote Apoptosis Induction by Hydroxyurea in PDAC Cells

We tested how hydroxyurea and entinostat affected murine PDAC cells from different mice [46,47]. These cells are named S821, 8296, 8248, and S411. We incubated them with the clinically relevant concentrations of 1 mM hydroxyurea and 1 µM entinostat [31,52], and additionally applied 5 µM entinostat to assess effects of higher doses of entinostat. After 24–48 h, we fixed the cells, stained them with PI, and analyzed their DNA contents by flow cytometry. Proliferating cells increase their DNA content from G1 phase (2N) to G2/M phase (4N). Dead cells with fragmented DNA have a DNA content below 2N (subG1 phase) [53]. DNA contents above 4N are found in cells that failed to segregate the replicated genomes in G2/M phase and underwent endomitosis [54].

**Figure 1:**
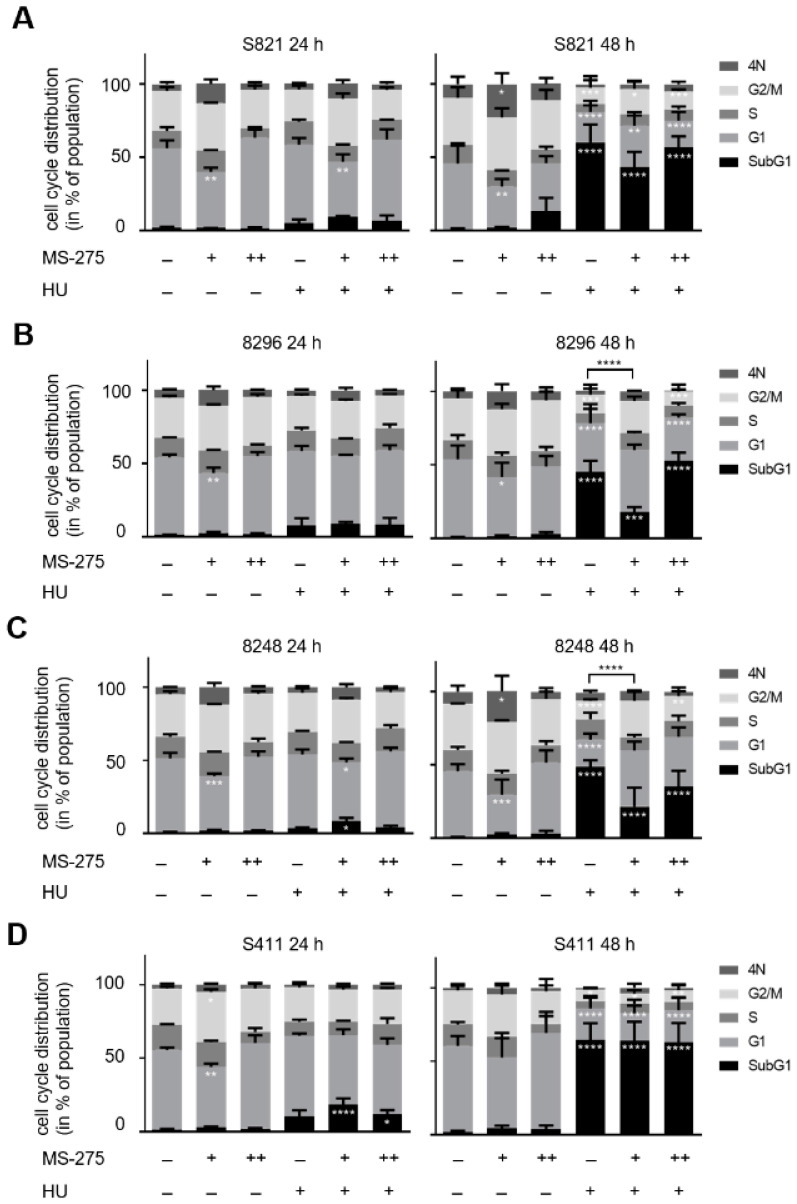

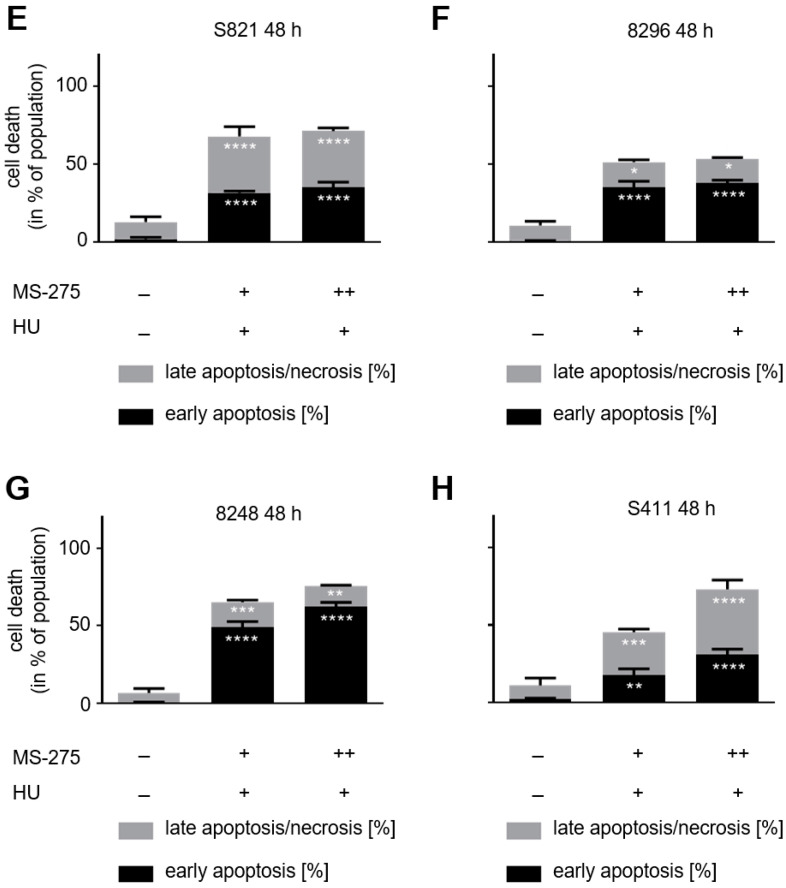
Apoptosis induction by hydroxyurea plus entinostat. PDAC cell lines were treated with various concentrations of entinostat (MS-275) and/or hydroxyurea (HU) for 24 h and 48 h. Cell cycle distribution and cell death were measured by flow cytometry of PI-stained cells. (**A**) S821, (**B**) 8296, (**C**) 8248, and (**D**) S411 cells were incubated for 24 h and 48 h with 1 µM (+), 5 µM entinostat (++), and/or 1 mM hydroxyurea (+). Data are mean ± SD values (24 h: *n* = 3; 48 h: *n* = 4). (**E**–**H**) The cells were treated with 1 µM entinostat (+) or 5 µM entinostat (++) plus 1 mM hydroxyurea (+) for 48 h and apoptosis was determined by flow cytometry for annexin-V/PI (*n* = 3). Data were statistically analyzed using one-way ANOVA (* *p* < 0.05, ** *p* < 0.01, *** *p* < 0.001, **** *p* < 0.0001).

After 24 h, 1 µM entinostat caused an accumulation of cells in G2/M phase but this effect vanished with 5 µM entinostat. Hydroxyurea decreased the G2/M phase populations of the four cell lines after 24 h. This is consistent with the inhibition of RRM2 by hydroxyurea, but its effects on G2/M phase did not reach statistical significance after 24 h. While the combination of 1 µM entinostat and 1 mM hydroxyurea increased the G2/M phase population in S821 cells, it decreased this phase in S411 cells. The combination of 5 µM entinostat and 1 mM hydroxyurea slightly reduced the G2/M and increased G1 phase populations. Addition of 1 µM entinostat to cells that were treated with 1 mM hydroxyurea had no impact (S821 and 8296 cells) or increased (8248 and S411 cells) the subG1 phase fractions that were induced by hydroxyurea in the PDAC cell lines. These effects were significant in 8248 and S411 cells (8248 cells, *p* = 0.0156; S411 cells, *p* < 0.0001). A total of 5 µM entinostat plus 1 mM hydroxyurea did not induce higher levels of subG1 fractions than hydroxyurea alone in all four cell lines. Moreover, 1 µM entinostat alone and in combination with 1 mM hydroxyurea increased the numbers of cells with DNA contents above 4N, but this did not reach statistical significance (Figure 1A–D).

After 48 h, 1 µM entinostat decreased the G1 phase populations and increased the numbers of cells with a DNA content above 4N in all cell lines. A total of 5 µM entinostat slightly increased the subG1 populations in S821 cells but did not alter cell cycle profiles significantly in the four cell lines. Furthermore, 1 mM hydroxyurea significantly increased the subG1 phase fractions in the cells (S821 cells: 60% (*p* < 0.0001), 8296 cells: 45% (*p* < 0.0001), 8248 cells: 48% (*p* < 0.0001), S411 cells: 65% (*p* < 0.0001)). Addition of 1 µM entinostat decreased the subG1 phase populations in the hydroxyurea treated PDAC cells S821 (43%, *p* < 0.0001), 8296 (18%, *p* = 0.0002), 8248 (21%, *p* < 0.0001). S411 cells still had a subG1 fraction of 64% (*p* < 0.0001) when 1 µM entinostat was given with 1 mM hydroxyurea. A total of 1 mM hydroxyurea plus 5 µM entinostat were more toxic than 1 mM hydroxyurea plus 1 µM entinostat in S821 cells (57%, *p* < 0.0001), 8296 cells (53%, *p* < 0.0001), and 8248 cells (35%, *p* < 0.0001). Moreover, 63% of S411 cells were in subG1 phase with this treatment (*p* < 0.0001) (Figure 1A–D; significances refer to control cells). A total of 1 µM entinostat significantly reduced hydroxyurea induced apoptosis after 48 h in 8296 and 8248 cells (Figure 1B,C; *p* < 0.0001).

An accumulation of cells in subG1 can indicate apoptosis as well as late apoptotic and necrotic cell death. To test whether the accumulation of subG1 phase cells is due to apoptosis, we stained the cells with annexin-V/PI and subjected them to flow cytometry. This method is a very sensitive indicator for apoptosis induction [53]. Annexin-V binds to phosphatidyl-serine which becomes surface-exposed in early apoptotic cells. The accumulation of PI indicates a breakdown of the cellular membrane potential during late apoptotic and necrotic cell death. Cells that cannot export PI and are annexin-V positive are late apoptotic and cells that only stain positive for PI are necrotic [53]. We found that 1 µM entinostat plus 1 mM hydroxyurea as well as 5 µM entinostat plus 1 mM hydroxyurea significantly induced apoptosis in the PDAC cells cell lines. While S821 and S411 cells were found more in late apoptosis, 8296 and 8248 cells were more in early apoptosis (Figure 1E–H).

These data demonstrate that therapeutically relevant doses of hydroxyurea significantly evoke apoptosis in the tested PDAC cells after 48 h. Unexpectedly, entinostat cannot pronounce this effect.

### 3.2. Hydroxyurea Induces Apoptosis More Effectively Than Irinotecan in the PDAC Cell Panel

**Figure 2:**
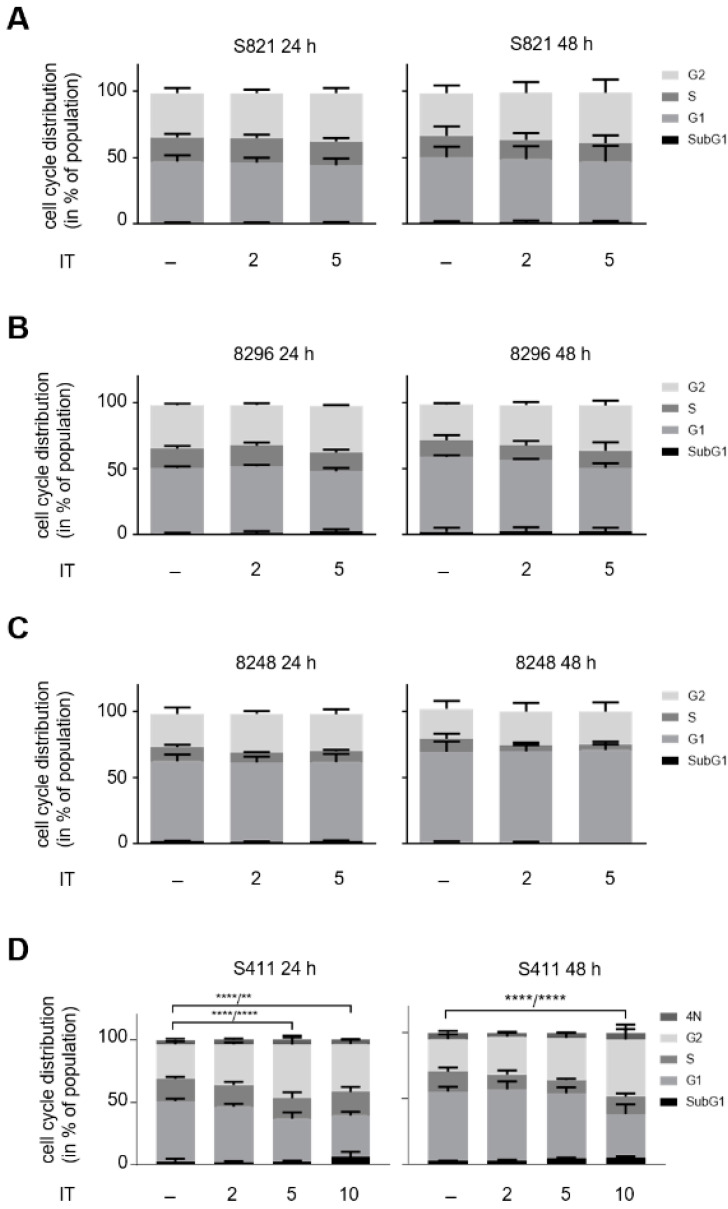

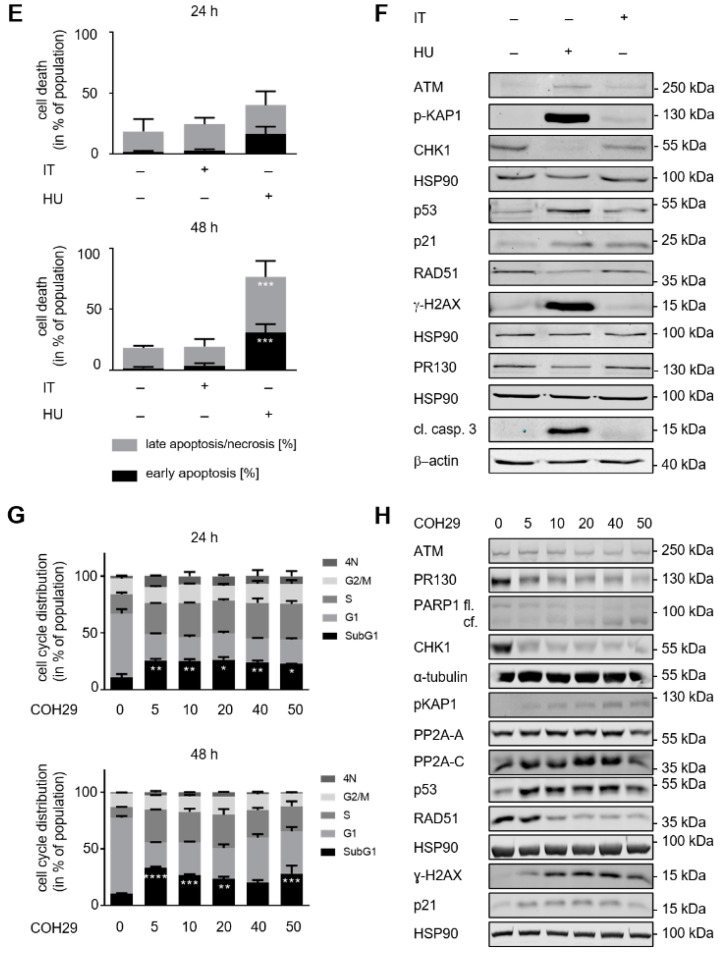
Hydroxyurea induces apoptosis and DNA damage in PDAC cell lines. PDAC cell lines were treated with irinotecan (IT) or hydroxyurea (HU). Cell cycle distributions and subG1 phases of (**A**) S821, (**B**) 8296, (**C**) 8248, and (**D**) S411 cells after incubation with 2, 5, and 10 µM irinotecan for 24 h and 48 h. The data were collected by flow cytometry of PI-stained cells and are shown as mean ± SD (*n* = 3; one-way ANOVA, * *p* < 0.05, ** *p* < 0.01, *** *p* < 0.001, **** *p* < 0.0001). The first *p*-values refer to G1 phase, the second *p*-values to G2/M phase. (**E**) Apoptosis analysis of S411 cells that were treated with 5 µM irinotecan or 1 mM hydroxyurea for 24 h and 48 h. Results were determined by flow cytometry by using annexin-V-FITC staining and are shown as mean ± SD (*n* = 3). Hydroxyurea induces significantly higher levels of apoptosis than irinotecan (apoptosis: 31% (*p* = 0.0005), late apoptosis/necrosis: 45% (*p* = 0.0005). Statistical analysis was done with two-way ANOVA (* *p* < 0.05, ** *p* < 0.01, *** *p* < 0.001, **** *p* < 0.0001). (**F**) S411 cells were treated with 5 µM irinotecan or 1 mM hydroxyurea for 24 h. The levels of ATM, p53, p21, RAD51, cleaved caspase-3 (cl. casp. 3) and PR130, as well as the phosphorylation of KAP1 and H2AX were measured by immunoblot. HSP90 and β-actin served as loading controls; *n* = 3. (**G**) A total of 5–50 µM COH29 were applied to S411 cells for 24–48 h. Flow cytometry determined cell cycle distributions and cells in subG1 phase; *n* = 2. (**H**) S411 cells were treated with 5–50 µM COH29 for 24 h. Immunoblot was done as indicated (fl., full-length PARP1; cf., cleaved form of PARP1). HSP90 and α-tubulin served as loading controls; *n* = 2.

Since irinotecan is frequently given to patients with PDAC [3], we assessed how it affects our cell panel. A total of 2–5 µM irinotecan dose- and time-dependently increased the percentages of cells in G2/M phase. Curiously, this was not associated with an increase in subG1 phase (Figure 2A–D).

Of the three cell lines, S411 cells responded most strongly and highly significant with a G2/M phase arrest to 5 µM irinotecan after 24 h. This was associated with a significantly decreased number of cells in G1 phase. A total of 10 µM were not more effective than 5 µM irinotecan, indicating a reached plateau (Figure 2D; *p* < 0.0001). This higher dose of irinotecan was though necessary to sustain the stalling of S411 cells in G2/M phase for 48 h (Figure 2D). Irrespective of the G2/M phase stalling, 10 µM irinotecan did not significantly increase the subG1 populations in such cells after 48 h (Figure 2D).

We chose S411 cells for additional analyses. We treated them with 5 µM irinotecan or 1 mM hydroxyurea for 24 h and analyzed apoptosis by flow cytometry for annexin-V/PI. While hydroxyurea significantly induced apoptosis in S411 cells, irinotecan did not cause apoptosis after 24 h and 48 h (Figure 2E).

We further analyzed these findings by immunoblot. Upon replication stress and DNA damage, checkpoint kinases are activated. These stabilize the tumor suppressor p53 by serine phosphorylation [55] and phosphorylate histone H2AX at S139 (ɣH2AX) [56]. Immunoblot analyses of lysates from S411 cells showed that hydroxyurea increased ATM levels, the phosphorylation of the ATM target KAP1 (p-KAP1), p53, and ɣH2AX. Moreover, we noted decreased levels of CHK1 and of the PP2A subunit PR130 (Figure 2F), which attenuates checkpoint kinase activation [32]. Concomitant with the increase of ɣH2AX, there was a downregulation of RAD51 (Figure 2F), which mediates DNA repair by homologous recombination [57]. Consistent with the increase in annexin-V/PI-positivity (Figure 2E), activated caspase-3 was detectable in S411 cells (Figure 2F). In contrast to hydroxyurea, irinotecan induced a slight accumulation of ATM and p-KAP1 (Figure 2F). Both hydroxyurea and irinotecan increased the levels of the p53 target gene p21 similarly, but this was not associated with an accumulation of p53 in irinotecan treated cells (Figure 2F).

To control these data, we treated S411 cells with the novel RNR inhibitor COH29, which inhibits RNR more avidly than hydroxyurea does [44]. COH29 dose-dependently stalled S411 cells in S phase after 24 h. After 48 h, the S phase arrest was maintained with up to 20 µM COH29. Higher doses stalled the cells in G1 phase after 48 h (Figure 2G). 5 µM COH29 significantly increased subG1 phase cells after 24 h. This was not augmented by higher doses or longer exposure times to this drug (Figure 2G).

At the biochemical level, COH29 evoked similar effects as hydroxyurea. COH29 dose-dependently reduced PR130, CHK1, and RAD51, increased p-KAP1, p53, ɣH2AX, and p21, and caused a dose-dependent apoptotic cleavage of PARP1 (Figure 2H).

These results show that hydroxyurea induces apoptosis, checkpoint kinases, and DNA damage. COH29 triggers similar processes as hydroxyurea, verifying their common target inhibition.

### 3.3. Hydroxyurea and Entinostat Dysregulate Proteins That Control the Cell Cycle and DNA Repair

**Figure 3:**
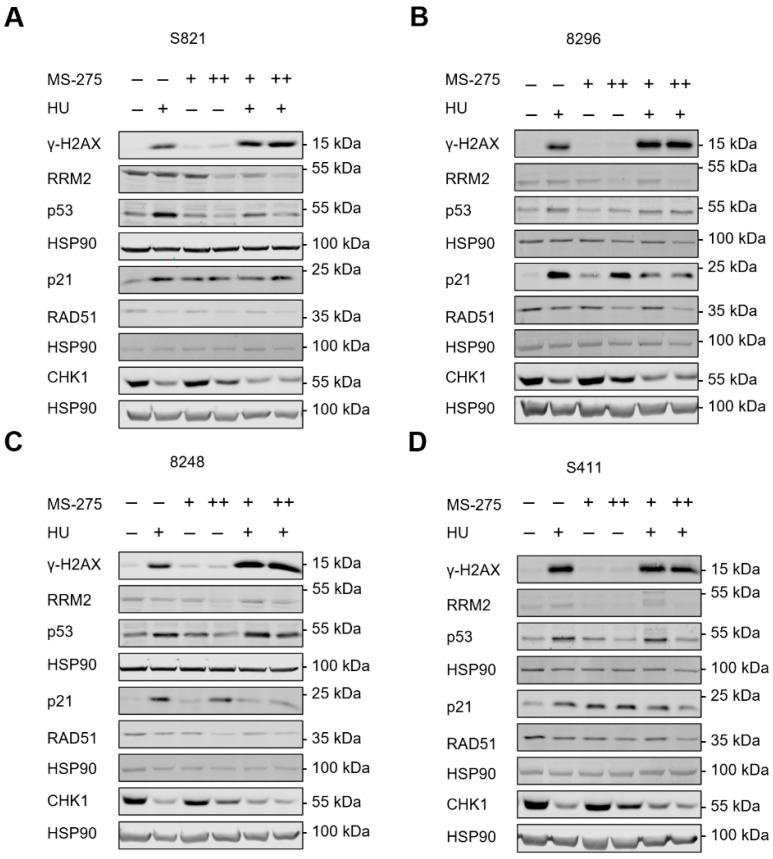
Immunoblot shows alterations in protein expression and posttranslational modifications in response to hydroxyurea and entinostat. (**A**–**D**) Mouse PDAC cell lines (S821, 8248, 8296, S411) were treated with 1 µM entinostat (+; MS-275), 5 µM entinostat (++; MS-275) and/or 1 mM hydroxyurea (+; HU) for 24 h. Immunoblot was done for the indicated proteins and HSP90 as loading control; *n* = 2.

Next, we analyzed DNA replication stress and DNA damage signaling in cells that were incubated with hydroxyurea and entinostat. Hydroxyurea induced an accumulation of the replication stress/DNA damage marker ɣH2AX in the four PDAC cell lines and 1 µM and 5 µM entinostat promoted this in S821, 8296, and 8248 cells (Figure 3A–C). Entinostat did not increase the hydroxyurea-induced levels of ɣH2AX in S411 cells (Figure 3D), which are the most hydroxyurea-sensitive of these cell lines (Figure 1D).

The single and combined application of hydroxyurea and entinostat reduced RAD51 and this effect was most pronounced in the cotreatment scheme of 1 mM hydroxyurea plus 5 µM entinostat. Inhibition of the RRM2 subunit of RNR stalls cell cycle progression, activates checkpoint kinases, and causes DNA damage if replication forks are stalled persistently [58]. A total of 5 µM entinostat dose-dependently reduced RRM2 and CHK1 in the four cell lines. We further found that hydroxyurea induced p53 and its target p21 in all four PDAC cell lines. Entinostat increased p21 but reduced the hydroxyurea-induced accumulation of p53 and p21 in S821 and S411 cells. Despite a reduction of p21, this was not the case for p53 in 8296 and 8248 cells that were exposed to hydroxyurea and entinostat (Figure 3A–D).

We conclude from these findings that entinostat modulates hydroxyurea-induced replication stress/DNA damage and the subsequent downstream signaling.

### 3.4. ATM Signaling Promotes Apoptotic DNA-Fragmentation in Hydroxyurea Treated PDAC Cells

**Figure 4:**
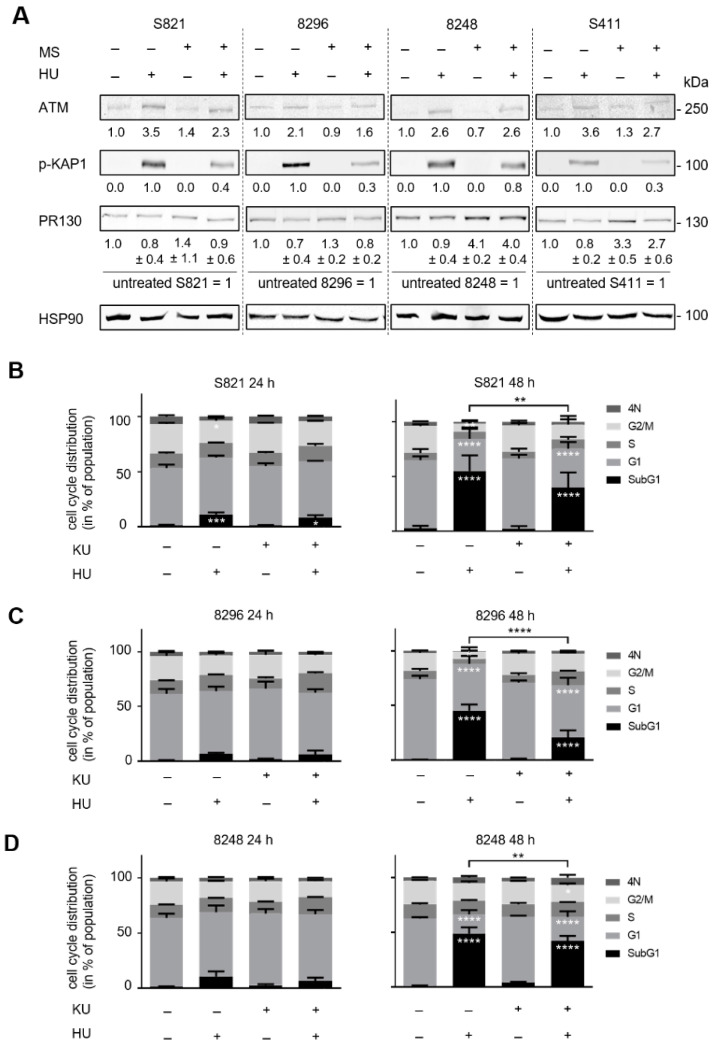

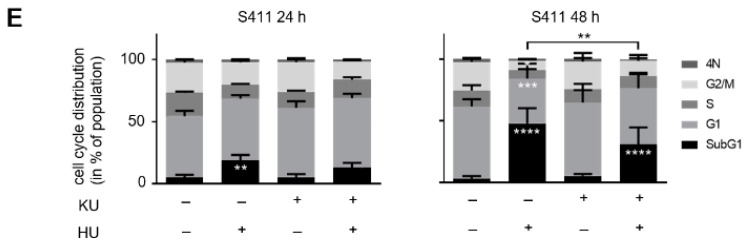
Flow cytometry data showing the impact of hydroxyurea and KU-60019 on cell cycle progression and apoptosis-associated DNA fragmentation. (**A**) The PDAC cell lines S821, 8296, 8248, and S411 were treated with 5 µM entinostat ± 1mM hydroxyurea for 24 h. PR130 as well as the phosphorylation of KAP1 were measured by immunodetection; HSP90 as loading control; *n* = 3. (**B**–**E**) The cells were treated with 1 mM hydroxyurea (HU) ± 5 µM KU-60019 (KU) for 24 h and 48 h; *n* = 3. Flow cytometry was carried out to measure cell cycle distributions and subG1 phase cells. Statistical analysis was done with two-way ANOVA (* *p* < 0.05, ** *p* < 0.01, *** *p* < 0.001, **** *p* < 0.0001).

We recently found that the HDAC1/HDAC2-regulated PP2A subunit PR130 binds and dephosphorylates pS1981-ATM in a PP2A holoenzyme complex in hydroxyurea treated human colon cancer cells [32]. In PDAC cells, hydroxyurea potently induces apoptosis and the phosphorylation of the ATM target protein KAP1. HDACi attenuate this and increase expression of PR130 after 24 h in 8248 and S411 cells (Figure 4A).

Since entinostat attenuated hydroxyurea induced apoptosis after 48 h (Figure 1), we speculated that ATM protected the cells from lethal effects of hydroxyurea. To test this, we applied the specific ATM inhibitor KU-60019 [59] together with hydroxyurea to the cells. KU-60019 had no notable effect on the four PDAC cell models after 24 h and 48 h. KU-60019 also had only a marginal impact on the hydroxyurea induced alterations of cell cycle progression after 24 h. In cells that we incubated with hydroxyurea and KU-60019 for 48 h, we noted that fewer cells accumulated in subG1 phase than in hydroxyurea treated cells. KU-60019 significantly reduced apoptosis induction by hydroxyurea in the tested cell lines (S821 (** *p* = 0.009), 8296 (**** *p* < 0.0001), 8248 (** *p* = 0.0069), S411 (** *p* = 0.0035)) after 48 h. Moreover, ATM inhibition in hydroxyurea treated cells was associated with an increase of cells in G1 phase and G2/M phase compared to hydroxyurea single treatment (Figure 4B–E).

These data illustrate that ATM is pro-apoptotic in hydroxyurea treated PDAC cells and this seems to preferentially affect PDAC cells in the G1 and G2/M phases.

### 3.5. ATM Signaling Is Necessary for Apoptosis Induction in Hydroxyurea Treated PDAC Cells

To corroborate the role of ATM, we assessed apoptosis markers by flow cytometry (annexin-V/PI) and immunoblot (cleaved caspase-3, cleaved PARP1). Moreover, we tested for ATM signaling (p-KAP1, p53) and ɣH2AX.

**Figure 5:**
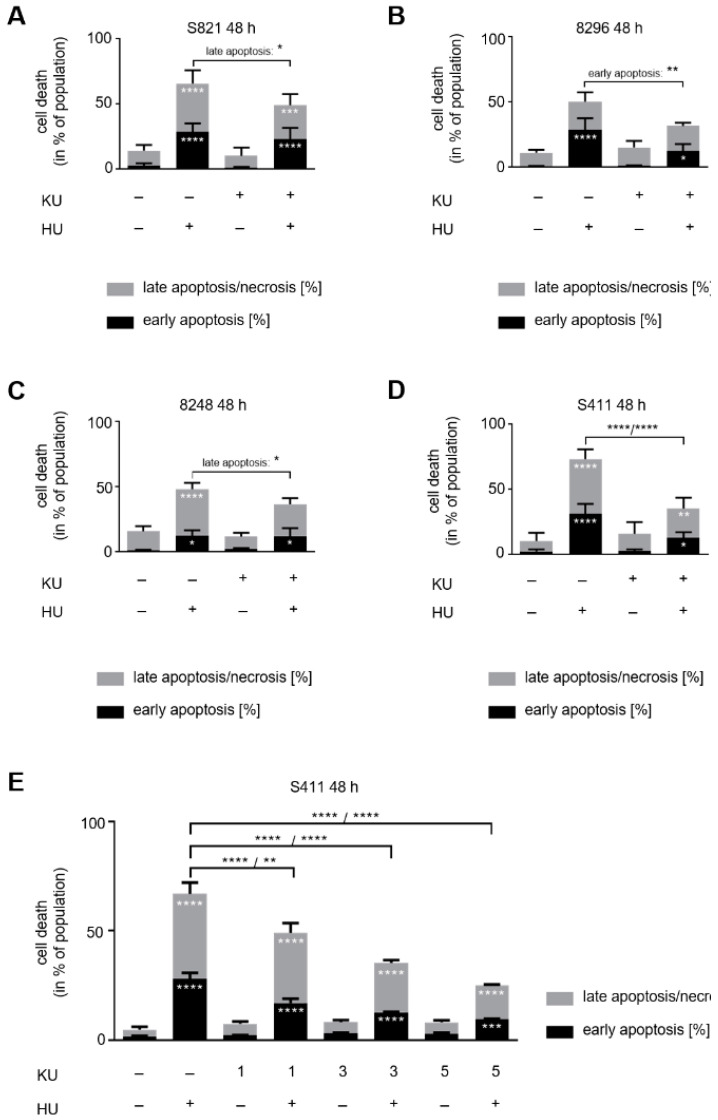

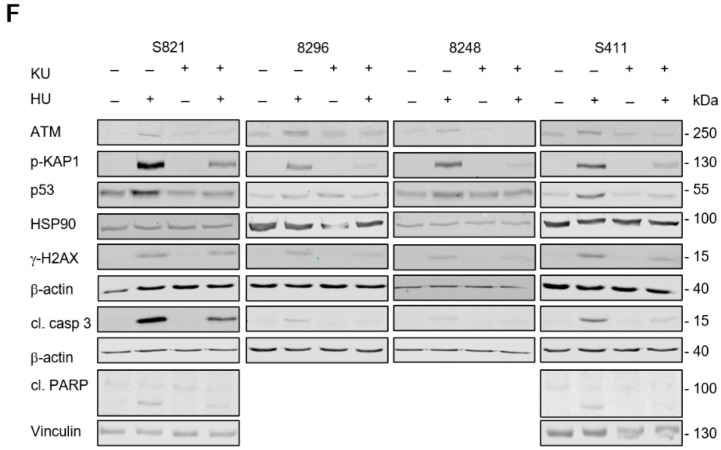
ATM inhibition counteracts apoptosis induction by hydroxyurea. Apoptosis analysis of (**A**) S821, (**B**) 8296, (**C**) 8248, and (**D**) S411 24 h after exposure to 1 mM hydroxyurea (HU) ± 5 µM KU-60019 (KU). Results were determined by flow cytometry using annexin-V-FITC staining and shown as mean ± SD (S821 *n* = 7; S411 *n* = 6; 8296/8248 *n* = 3). Statistical analysis was done with two-way ANOVA (* *p* < 0.05, ** *p* < 0.01, *** *p* < 0.001, **** *p* < 0.0001). (**E**) S411 cells were treated with 1 mM hydroxyurea and 1–5 µM of KU-60019. Results were collected by flow cytometry using annexin-V-FITC staining and are shown as mean ± SD (*n* = 3). (**F**) Immunoblot was done as indicated, with lysates from the four cell lines that were treated as mentioned above; fl., full-length PARP1; cf., cleaved form of PARP1; cl. casp. 3, cleaved form of caspase-3; HSP90, β-actin, vinculin as loading controls (S821 *n* = 3; 8296/8248/S411 *n* = 2).

In PDAC cells that we exposed to hydroxyurea for 24 h, KU-60019 reduced apoptosis induction by hydroxyurea in 8296 and S411 cells (Supplementary Figure S1). After 48 h, KU-60019 reduced apoptosis induction by hydroxyurea in all four PDAC cell lines. KU-60019 as a single drug had no impact on the cells (Figure 5A–D). In S821 cells, apoptosis induction by hydroxyurea was reduced from 66% to 49% by KU-60019 (*p* = 0.0191 for late apoptosis); in 8296 cells, apoptosis induction by hydroxyurea was reduced from 50% to 32% by KU-60019 (*p* = 0.0059 for early apoptosis); in 8248 cells, apoptosis induction by hydroxyurea was reduced from 48% to 36% by KU-60019 (*p* = 0.0169 for late apoptosis); in S411 cells, apoptosis induction by hydroxyurea was reduced from 73% to 35% by KU-60019 (*p* < 0.0001) (Figure 5A–D).

In ɣ-irradiated cells, KU-60019 specifically inhibits ATM at up to ≥10 µM [59]. To test whether this holds for hydroxyurea-induced ATM, we incubated S411 cells with 1 mM hydroxyurea ± 1/3/5 µM KU-60019. Already 1 µM KU-60019 significantly attenuated pro-apoptotic effects of hydroxyurea (Figure 5E), indicating that we specifically analyzed ATM-dependent effects with KU-60019.

Immunoblot analyses confirmed that KU-60019 acted on-target and suppressed ATM-dependent replication stress signaling. KU-60019 blocked the hydroxyurea induced phosphorylation of KAP1 and H2AX and the accumulation of ATM and p53 in the four cell lines. KU-60019 also attenuated the activation of caspase-3 in these cells. S821 and S411 cells are more sensitive to apoptosis induction by hydroxyurea and accumulate more activated caspase-3 than 8296 and 8248 cells. This ties in with a detectable cleavage of PARP1 in S821 and S411 cells (Figure 5F).

These data illustrate that ATM promotes apoptosis induction by hydroxyurea in PDAC cells.

## 4. Discussion

We found that hydroxyurea and COH29 induced apoptosis and an accumulation of the tumor suppressor p53 and its target p21 in murine PDAC cells. In contrast to this, hydroxyurea increased the transcription of the *p21* mRNA p53-dependently, but this did not translate into an accumulation of the p21 protein in human solid tumor-derived and leukemic cancer cells [32,33,51,60]. This can be explained by a CHK1-dependent suppression of *p21* mRNA translation [61]. In the PDAC cell panel, hydroxyurea strongly reduced CHK1, which could explain why p21 accumulates in such cells when they are treated with hydroxyurea. The reduction of CHK1 can in turn be explained by a p53-dependent suppression of *CHK1* mRNA expression [62]. Unfortunately, we could not detect the phosphorylation of CHK1 at S296/317/345 in mouse cells with a set of commercially available antibodies (data not shown) but it is well-established that hydroxyurea triggers the phosphorylation of CHK1 by replication stress activated ATR [32,58]. Since CHK1 stalls cell cycle progression upon replication stress [63], the depletion of CHK1 by hydroxyurea in PDAC cells could be the reason why these are not arrested in S phase by this drug. Hydroxyurea and COH29 target RRM2, which is required for S phase entry and progression [30]. This consequently leads to a decrease of G2/M phase cells. COH29 which is a stronger RNR inhibitor than hydroxyurea [44,45] delays cell cycle progression of PDAC cells in S phase. This could be interpreted in a way that the strength of RNR inhibition determines the type of cell cycle arrest. A not mutually exclusive explanation is that an apoptosis associated loss of S phase cells upon treatment with hydroxyurea prevents an increase in the S phase population despite RRM2 inhibition. It is additionally possible that an incomplete suppression of cell cycle progression by hydroxyurea [64] is more pro-apoptotic than a strong inhibition of RRM2 that stalls cells in S phase.

This work further shows a strong, pro-apoptotic activation of ATM by hydroxyurea in PDAC cells. We measured this as p-KAP1 because our antibodies were not able to detect p-ATM in murine PDAC cells (data not shown). The ATM-related checkpoint kinase ATR is one of the first molecules that are activated upon dNTP depletion by hydroxyurea [58,65,66]. Activated ATR and the single strand DNA binding protein RPA protect stalled replication forks and prevent DNA collapse and double strand breaks [58]. DNA repair proteins such as RAD51 [67] and BRCA2 [68] contribute to the stability and repair of DNA replication forks that are endangered by dNTP depletion. Accordingly, the ATR-CHK1 axis is a key survival factor in hydroxyurea treated cancer cells from colon, lung, thyroid, and skin [32,69,70]. ATM can also protect colorectal cancer cells and lymphoblastoid cells from hydroxyurea-induced cell death [32,71], but seems less effective than ATR [32]. While ATM is not required to stabilize p53 in hydroxyurea treated lymphoblastoid cells [71], we see that p53 stabilization depends on ATM in hydroxyurea treated PDAC cells. This agrees with the stabilization of p53 by checkpoint kinase dependent phosphorylation [55]. ATM is equally necessary for the accumulation of the replication stress/DNA damage marker ɣH2AX in response to hydroxyurea. These data verify that ATM induces key replication stress signaling pathways in murine PDAC cells. Thus, unlike in many other cell systems, ATM is necessary for apoptosis induction by hydroxyurea in these PDAC cells.

We collected our data on a role of ATM in hydroxyurea treated PDAC cells with 1–5 µM KU-60019. This compound inhibits the ATM kinase in vitro with an IC_50_ of 6.3 nmol/L and KU-60019 has little activity against the ATM-related checkpoint kinases DNA-PKcs and ATR (IC_50_ 1.7 μM to >10 μM). Moreover, 10 µmol/L KU-60019 did not sensitize cells lacking ATM to DNA damage induced cell death [59]. These data suggest that we have specifically investigated ATM in our assays. The notion that an increased activation of ATM promotes apoptosis induction by hydroxyurea in PDAC cells is in line with our recent finding that a genetic elimination of the phosphatase-2A subunit PR130 prevents the entinostat-induced dephosphorylation of ATM and that this sensitizes colorectal cancer cells to apoptosis induction by hydroxyurea plus entinostat. Entinostat not only induces PR130 expression but also its acetylation [32]. Although we see only a weak induction of PR130 in some PDAC cells by entinostat, it clearly reduced hydroxyurea induced p-KAP1. We have not investigated PR130 acetylation in PDAC cells, but it is possible that the acetylation of PR130 critically determines the ATM (de)phosphorylation state in such cells.

ATM as well as CHK1 are downstream targets of ATR in cells that are exposed to hydroxyurea [72]. Together with the clear activation of ATM and ɣH2AX, we detected a loss of CHK1 in the hydroxyurea treated PDAC cells. This is reminiscent of cells in which ATR and CHK1 are blocked. Such cells have a strong activation of ATM, DNA double strand breaks, unscheduled DNA replication origin firing, cell cycle progression despite stress, and eventually an induction of apoptosis [32,58,65,69,70,73].

We additionally demonstrate that the inhibition of HDAC1, HDAC2, and HDAC3 with entinostat attenuates RRM2 and RAD51 in PDAC cells. These data confirm previous reports on a suppression of RAD51 by HDAC inhibition [73]. The reduction of RRM2 by 5 µM entinostat likely enhances the inhibitory effect of hydroxyurea on RRM2. Moreover, the reduction of RAD51 by entinostat and the combination treatment with hydroxyurea can explain the increased accumulation of ɣH2AX and p-KAP1, which are ATM targets and markers for pronounced DNA replication stress and DNA breaks [56,73]. Since direct inhibition of ATM in hydroxyurea treated PDAC cells even reduced the DNA damage marker ɣH2AX, we assume that reduction of the DNA repair protein RAD51 by entinostat rather than inactivation of ATM causes the DNA damage phenotype in PDAC cells that are exposed to hydroxyurea plus entinostat.

We used hydroxyurea to cause replication stress due to a general stalling of replication forks and tested how the inhibition of ATM affects cell fate upon such conditions. Oncogenes like mutated RAS cause DNA replication stress due to increased cell proliferation [74] and loss-of-function mutations in the DNA repair proteins BRCA1, BRCA2, ATM, and PALB2 occur in inherited and sporadic PDAC cells [75]. Mouse models with an expression of an oncogenic mutant of the small G protein RAS [76] and a deletion of ATM in the pancreas mimic human PDAC [77]. Like in our cell model with drug-induced DNA replication stress, the deletion of ATM increased DNA damage foci in murine PDACs with mutant RAS. This is associated with a reduced DNA repair capacity by homologous recombination. Furthermore, loss of ATM is associated with a shorter survival of PDAC patients and of mice with PDAC [77]. Thus, this increased DNA damage does not translate into increased cancer cell death, but rather into poor survival. These findings correspond to our notion that while entinostat suppressed ATM signaling and increased DNA damage, it did not augment apoptosis induction by hydroxyurea in PDAC cells. We therefore conclude that a loss of ATM-dependent pro-apoptotic effects seems to be more critical than its impact on DNA integrity in PDAC cells with RNR inhibition.

## 5. Conclusions

Globally, PDAC remains an unsolved clinical problem. We show that hydroxyurea induces DNA damage and apoptosis in PDAC cells. In contrast to other cell systems, ATM is pro-apoptotic in hydroxyurea treated PDAC cells.

## Data Availability

All data are available in the manuscript.

## Supplementary Materials

The following are available online at https://www.mdpi.com/article/10.3390/cells10102520/s1, Figure S1: Impact of hydroxyurea and KU-60019 on PDAC cells after 24 h.
