# Supplementary material for: Class 1 Histone Deacetylases and Ataxia-Telangiectasia Mutated Kinase Control the Survival of Murine Pancreatic Cancer Cells upon dNTP Depletion
Source: Cells. 2021 Sep 23;10(10):2520. doi: 10.3390/cells10102520 (PMC8534202; doi:10.3390/cells10102520)
Supplement: Supplementary file 1 [file cells-10-02520-s001.zip › cells-1317266-supplementary.pdf]

Supplementary Material

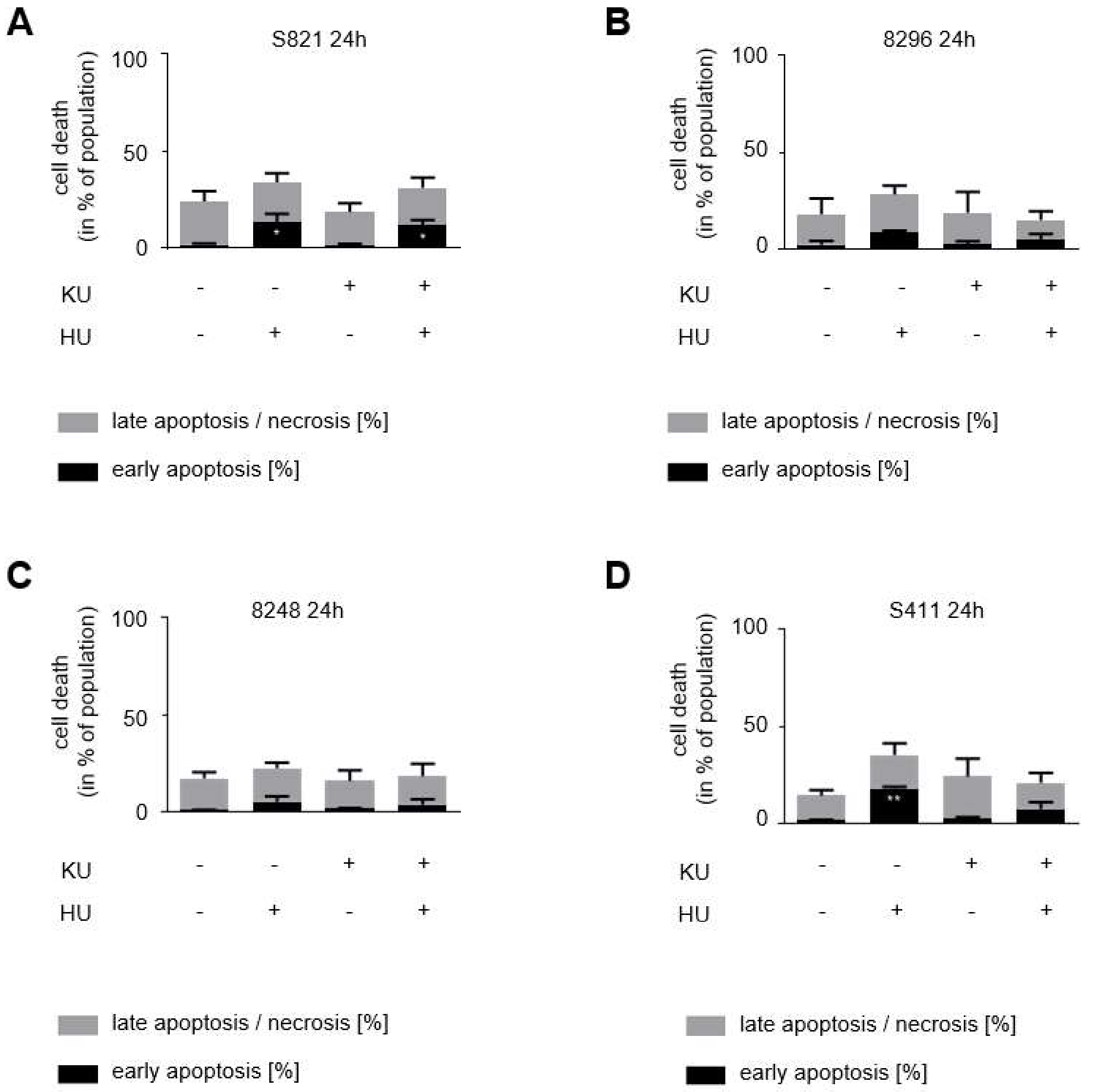

Figure S1. Impact of hydroxyurea and KU-60019 on PDAC cells after 24 h. Apoptosis analysis of (A) S821, (B) 8296, (C) 8248 and (D) S411 24 h after a treatment with 5 µM KU-60019 ± 1 mM hydroxyurea. Results were determined by flow cytometry using annexin-V-FITC staining and are shown as mean ± SD (n=3). The differences in annexin-V positive cells were statistically analyzed using one-way ANOVA (*p<0.05, **p<0.01).
